# Unintentional Injuries among Psychiatric Outpatients with Major Depressive Disorder

**DOI:** 10.1371/journal.pone.0168202

**Published:** 2016-12-16

**Authors:** Ching-I Hung, Chia-Yih Liu, Ching-Hui Yang

**Affiliations:** 1 Department of Psychiatry, Chang-Gung Memorial Hospital at Linkou, Taoyuan, Taiwan; 2 Chang-Gung University School of Medicine, Taoyuan, Taiwan; 3 Department of Nursing, Chang Gung Institute of Technology, Tao-Yuan, Taiwan; University of Illinois at Chicago College of Medicine, UNITED STATES

## Abstract

**Background:**

No study has investigated the percentages of and factors related to unintentional injuries among psychiatric outpatients with major depressive disorder (MDD). This study aimed to investigate these issues.

**Methods:**

One-hundred and forty-one outpatients with MDD at baseline were enrolled from psychiatric outpatients by systematic sampling, and 119 subjects attended a one-year follow-up. Self-reported unintentional injuries in the past one year were recorded. Psychiatric disorders were diagnosed using the Structured Clinical Interview for DSM-IV-TR. The severity of depression was evaluated by the Hamilton Depression Rating Scale. Other data, including body weight and height, cigarette smoking, headaches, and medications, were collected. Generalized Estimating Equations were used to investigate independent factors related to unintentional injuries.

**Results:**

At baseline and follow-up, 40.4% and 27.7% of subjects had experienced at least one unintentional injury in the past one year, respectively. About half of subjects with unintentional injuries needed medical treatment for injuries and had functional impairment due to injuries. A greater severity of depression, cigarette smoking, a higher body mass index, and an older age were independent risk factors related to unintentional injuries.

**Conclusion:**

Unintentional injuries that increased the medical burden and functional impairment were common among outpatients with MDD and should not be neglected. Treatment of depression, control of body weight, and quitting cigarettes might be helpful to prevent unintentional injuries.

## Background

Unintentional injuries (UI) cause a burden to society because they may lead to mortality and disability [1]. Psychological distress and mental disorders are related to UI [2,3]. Previous studies have reported that depression is an important factor related to UI in different populations, including children, adolescents, retired employees, geriatrics, and the general population [4–10]. Compared with subjects without depression, subjects with depression have a significantly higher risk of UI in the general population [5]. Moreover, depression was found to be related to prolonged activity loss after injury [5]. In a cohort study, subjects with depression were found to have a 41% increased risk of UI after controlling for confounding factors [7].

Although many studies have reported that depression is an important factor related to UI, no study, to the best of our knowledge, has investigated the percentages and risk factors of UI among psychiatric outpatients with major depressive disorder (MDD). In fact, previous studies related to the issue of injuries among patients with MDD have mostly focused on self-destructive and suicidal behaviors [11]. Prevention of UI among outpatients with MDD should be as important as prevention of self-destructive and suicidal behaviors, because both intentional and unintentional injuries cause functional impairment and increase the burden on medical care and on society in general. Understanding the percentages and factors related to UI is the first step in the prevention of UI among outpatients with MDD.

Therefore, this study aimed to investigate the percentages of and factors related to UI among psychiatric outpatients with MDD at baseline and one-year follow-up. We hypothesized that UI were common among these patients and that the severity of depression was an important factor related to UI after controlling for other confounding factors.

## Methods

### Subjects

This study was approved by the Institutional Review Board of the Chang Gung Memorial Hospital at Linkou, a medical center in Taiwan, and was conducted in the psychiatric outpatient clinic of the same hospital from August 2008 to July 2010. In the first step, the medical charts of psychiatric outpatients within the age range of 20 to 60 years were reviewed. Outpatients with the following conditions as identified in the medical charts were excluded: 1) patients with psychotic disorders or symptoms, including schizophrenia, schizoaffective disorder, delusional disorder, other psychotic disorders, and mood disorders with active psychotic symptoms; 2) patients with dementia, delirium, mental retardation, and mental disorders due to general medical diseases or substance use.

In the second step, outpatients who did not meet the above two exclusion conditions were assigned a number. Then, systematic sampling was used to draw one subject from 5 outpatients. Systematic sampling is a random sampling technique by which sample subjects from a larger group of patients are selected according to a random starting point and a fixed periodic interval. In this study, the starting point was three, and the fixed periodic interval was five. In the third step, the outpatients selected by systematic sampling were interviewed based on the Structured Clinical Interview for DSM-IV-text revision Axis I Disorders (SCID) [12,13]. MDD, substance abuse or dependence, anxiety disorders, including panic disorder and/or agoraphobia, social phobia, specific phobia, obsessive-compulsive disorder, post-traumatic stress disorder, and generalized anxiety disorder, were diagnosed. Patients with MDD were considered eligible subjects in this study.

The two exclusion criteria in the first step excluded outpatients with a compromised capacity to consent. Therefore, the enrolled subjects with MDD had the capacity to consent. Written informed consent, based on the guidelines regulated in the Declaration of Helsinki, was obtained from all subjects before study enrollment.

### Evaluation of unintentional injuries

Subjects were interviewed for evaluation of UI in the past one year. The investigators emphasized that self-destructive acts or suicidal attempts were not considered as UI. The types of UI in the past one year, including falling down on the ground, falling from height, traffic accidents, scalds or burns, and other types, were recorded. Other data related to UI were also collected, including 1) whether medical or surgical treatment after the UI was indicated; 2) how long the patient had functional impairment due to UI in terms of their occupation, domestic work, or daily activities.

### Evaluation of depression, sleep, and other factors related to unintentional injuries

The Hamilton Depression Rating scale (HAMD) was used to evaluate the severity of depression in the past week [14]. The Chinese version of the Pittsurgh Sleep Quality Index (CPSQI)^20^ for the assessment of the severity of sleep disturbance. the Pittsburgh Sleep Quality Index (PSQI), a self-rated questionnaire to assess seven components of sleep, was used to evaluate sleep disturbance in the past month [15,16]. Higher scores in the HAMD and PSQI indicated worse depression and a poorer sleep quality, respectively.

The patients were divided into three depressive states in the index month of the investigation. A current major depressive episode (MDE), indicated by fulfillment of the criteria of a MDE based on the DSM-IV-text revision, was diagnosed based on the SCID. Full remission of depression was defined as a HAMD score ≤7. Partial remission of depression was identified when the severity of depression was of a status between that of a current MDE and full remission.

A structured headache intake form, which was designed to meet the operational criteria of the *International Classification of Headache Disorders*, 2nd edition (ICHD-2) [17], was administered. An investigator experienced in headache interviewed all subjects after they had completed the headache intake form and made headache diagnoses based on the ICHD-2. The visual analog scale (VAS) was used to evaluate the average intensity of headache in the past week. Headache was investigated because severe headache is related to impaired cognitive function [18].

Five demographic variables (age, sex, years of education, marital status, and employment status), a regular habit of cigarette smoking in the past one year, and the kinds and dosages of medications in the index month of the investigation were recorded based on medical charts. Body height and weight were measured using standard instruments, and body mass index (BMI) was calculated.

### Procedure

After enrollment, subjects were evaluated in terms of the above parameters or indices. This neutral clinical study did not control medications during the study period. After the baseline evaluation, the subjects were followed-up one year later. The above parameters or indices were re-evaluated and recorded at the one-year follow-up.

### Statistical methods

All statistical analyses were performed using SPSS for Windows 20.0. The independent t test, paired t test, chi-square test, Mann-Whitney test, Kruskal-Wallis test, Wilcoxon test, and McNemar test were used where appropriate. Generalized Estimating Equations (GEE) with logit link were used to identify independent factors related to UI. The dependent variable was the presence or absence of UI. Among the 17 related variables, which included the five demographic variables, HAMD score, PSQI score, intensity of headache, BMI, migraine (yes or no), anxiety comorbidities (yes or no), cigarette smoking (yes or no), alcohol abuse or dependence (yes or no), use of antidepressants (yes or no), use of antipsychotics (yes or no), use of any benzodiazepine or non-benzodiazepine hypnotic (yes or no), and time point (follow-up *vs*. baseline), only variables with *p* < 0.1 in the univariate tests were placed into the GEE model as independent variables.

Moreover, multiple logistic regressions with Wald and forward selection were employed to identify independent factors at baseline to predict subjects with UI both at baseline and follow-up. The independent factors included all of the above 17 factors at baseline, with the exception of time point.

A two-tailed *p* value < 0.05 was considered statistically significant in all statistical analyses.

## Results

### Subjects

A total of 2201 patients were screened during the study period and 1054 patients were excluded due to psychotic disorders (475 patients), mental disorders due to general medical conditions or substance use (229 patients), dementia or aged > 60 years (198 patients), mental retardation (77 patients), aged < 20 years (62 patients), and other exclusion criteria (13 patients). The other 1147 subjects participated in systematic sampling, and 229 patients were selected, then diagnosed using the SCID. The 229 patients consisted of 141 patients with MDD, 15 with bipolar I disorder, 16 with bipolar II disorder, 36 with only anxiety disorders, and 21 without the above mood or anxiety disorders. Among the 141 patients with MDD (S1 Dataset), 119 (84.4%) patients agreed to participate in the one-year follow-up. There were no significant differences in the five demographic variables at baseline between subjects with (*n* = 119) and without (*n* = 22) a one-year follow-up period (with follow-up *vs*. without follow-up: age: 44.0 ± 9.4 *vs*. 43.1 ± 11.4 years; educational years: 11.3 ± 3.3 *vs*. 10.3 ± 3.0; percentage of subjects of the female gender, those with paid employment, and married subjects: 67.2 *vs*. 68.2, 58.8 *vs*. 59.1, and 66.4 *vs*. 72.7, respectively). Table 1 shows the differences in demographic variables between the patients with and without UI. There were no significant differences in the five demographic variables at baseline and follow-up, with the exception of the fact that subjects with UI were of an older age at baseline.

**Table 1:** Differences in demographic variables between patients with and without unintentional injuries.

	Unintentional injuries at baseline			Unintentional injuries at one-year follow-up		
	Full sample (*n* = 141)	Yes (*n* = 57)	No (*n* = 84)	Full sample (*n* = 119)	Yes (*n* = 33)	No (*n* = 86)
Age (years)	43.9 ± 9.7	45.8 ± 8.7*	42.6 ± 10.1	45.0 ± 9.4	46.9 ± 9.0	44.3 ± 9.5
Years of education	11.1 ± 3.3	10.6 ± 3.2	11.5 ± 3.3	11.3 ± 3.3	10.5 ± 3.3	11.6 ± 3.3
Gender (female, %)	67.4	68.4	66.7	67.2	75.8	64.5
Paid employment (yes, %)	58.9	49.1	65.5	58.8	51.5	61.6
Married (yes, %)	67.4	71.9	64.3	66.4	63.6	67.4

*p < 0.05

Among the 141 patients, 90 (63.8%) subjects had at least one anxiety comorbidity, including 34 (24.1%) subjects with panic disorder, 20 (14.2%) with agoraphobia, 28 (19.9%) with social phobia, 48 (34.0%) with specific phobia, 17 (12.1%) with post-traumatic stress disorder, 9 (6.4%) with obsessive-compulsive disorder, and 2 (1.4%) with generalized anxiety disorder. Thirty-one (22.0%) subjects had alcohol abuse or dependence and five (3.5%) had other substance abuse or dependence. Fifty-nine (41.8%) subjects had migraine headache. Subjects with other substance abuse or dependence were not included in further analysis because of the small sample size.

At baseline, 50 subjects (35.5%) had a history of a suicidal attempt. Compared with MDD patients without UI at baseline, a higher percentage of MDD patients with UI had a history of suicidal attempt (47.4% *vs*. 27.4%, *p* < 0.01).

### Types and impacts of unintentional injuries

Table 2 shows the types and percentages of UI. Falling down on the ground was the most common type of UI, followed by traffic accidents, both at baseline and follow-up.

**Table 2:** Types and percentages of unintentional injuries.

	Baseline (*n* = 141)	One-year follow-up (*n* = 119)
	Number (%)	Number (%)
Any unintentional injury	57 (40.4%)	33 (27.7%)
Falling down on the ground	32 (22.7%)	19 (16.0%)
Traffic accident	20 (14.2%)	14 (11.8%)
Scald or burn	9 (6.4%)	2 (1.7%)
Fall from height	7 (5.0%)	2 (1.7%)
Other unintentional injury	9 (6.4%)	5 (4.2%)

At baseline, 57 (40.4%) subjects had at least one UI in the past one year. Among them, 32 (56.1%) subjects reported that medical or surgical treatment was indicated for their UI, including 17 subjects treated in outpatient clinics, 8 in the emergency room, and 7 requiring admission (duration of admission: 9.1± 8.2 days). Moreover, 28 (49.1%) subjects reported functional impairment in terms of their occupation, domestic work, or daily activities due to UI (duration of functional impairment: 44.4 ± 95.5 days).

Compared with patients without MDD (*n* = 57, including 36 with anxiety disorders only and 21 without mood or anxiety disorders), a higher percentage of patients with MDD had UI at baseline (40.4% *vs*. 17.5%, *p* < 0.01).

At the one-year follow-up, 33 (27.7%) subjects had at least one UI. Among them, treatment was indicated for UI in 18 (54.5%) subjects, including 7 subjects treated in outpatient clinics, 9 in the emergency room, and 2 requiring admission (duration of admission: 17.0 ± 18.4 days). Moreover, 15 (45.5%) subjects had functional impairment due to UI (duration of functional impairment: 34.2 ± 60.4 days). Among the 119 subjects, 20 (16.8%) had UI at both baseline and follow-up, 31 (26.1%) had UI at baseline but not at follow-up, 13 (10.9%) did not have UI at baseline but did at follow-up, and 55 (46.2%) did not have UI either at baseline or follow-up.

Compared with the percentage of UI at baseline, the percentage of UI at follow-up was significantly decreased (40.4% *vs*. 27.7%, *p* = 0.01).

### Differences in factors related to UI between groups

Table 3 shows the differences in factors related to UI between groups. At baseline, patients with UI had a greater severity of depression, poorer sleep quality, and higher percentage of cigarette smoking as compared with patients without UI. At follow-up, patients with UI had a greater severity of depression, poorer sleep quality, a higher BMI, and a higher percentage of cigarette smoking as compared with patients without UI. There were no significant differences in the percentages of migraine, anxiety comorbidities, alcohol abuse or dependence, use of antidepressants, antipsychotics, and benzodiazepine and/or non-benzodiazepine hypnotics, at baseline and follow-up.

**Table 3:** Differences in scores and percentages between patients with and without unintentional injuries.

	Unintentional injuries at baseline			Unintentional injuries at one-year follow-up		
	Total sample (*n* = 141)	Yes (*n* = 57)	No (*n* = 84)	Total sample (*n* = 119)	Yes (*n* = 33)	No (*n* = 86)
BMI	24.0 ± 3.9	24.4 ± 4.2	23.6 ± 3.7	24.1 ± 4.0	26.2 ± 4.3**	23.3 ± 3.6
HAMD	13.7 ± 6.9	15.5 ± 7.0*	12.5 ± 6.6	11.1 ± 7.4	14.1 ± 8.3*	10.0 ± 6.7
PSQI	11.9 ± 3.8	12.9 ± 3.9**	11.3 ± 3.6	11.1 ± 4.5	12.4 ± 5.0*	10.6 ± 4.1
Headache intensity	2.9 ± 3.1	3.4 ± 3.2	2.6 ± 3.1	2.8 ± 2.9	3.6 ± 2.9	2.4 ± 2.8
Cigarette smoking (yes; %)	23.4	35.1**	15.5	25.2	42.4**	18.6
Anxiety disorders (yes; %)	63.8	64.9	63.1	63.9	51.5	68.6
Migraine (yes; %)	41.8	45.6	39.3	42.0	45.5	40.7
Alcohol abuse or dependence (yes; %)	22.0	24.6	20.2	21.0	27.3	18.6
Antipsychotics (yes; %)	22.7	26.3	20.2	16.8	21.2	15.1
Anxiolytics or hypnotics (yes; %)	95.0	94.7	95.2	79.8	84.8	77.9
Antidepressants (yes; %)	95.7	96.5	95.2	76.5	87.9	72.1

*p < 0.05;

**p <0.01

HAMD = Hamilton Depression Rating scale; PSQI = Pittsburgh Sleep Quality Index; BMI = body mass index.

At baseline and follow-up, the percentages of UI in the three depressive state groups differed significantly, being 56.1% (23/41) and 52.4% (11/21) for a current MDE, 38.4% (28/73) and 27.3% (15/55) for partial remission of depression, and 22.2% (6/27) and 16.3% (7/43) for full remission of depression, respectively.

At the follow-up, the HAMD and PSQI scores significantly decreased, and changes in headache intensity and BMI were not significant. At the follow-up, 21(17.6%) subjects did not accept pharmacotherapy at the index month of investigation. There was no significant difference (*p* = 0.43) in the percentage of UI between those who accepted pharmacotherapy (29.6%; 29/98) and those who did not (19.0%; 4/21).

### Factors independently predicting UI

Table 4 shows the independent factors related to UI. After controlling for the effects of time point, the HAMD score, cigarette smoking, BMI, and age remained significant independent factors related to UI. The HAMD score was the most significant factor.

**Table 4:** Factors independently associated with unintentional injuries.

	Odds ratio	95% Confidence interval	*p*-value
HAMD	1.07	1.03–1.12	0.002
Cigarette smoking	2.80	1.42–5.54	0.003
BMI	1.12	1.04–1.20	0.004
Age	1.03	1.00–1.07	0.039
Time point (follow-up *vs*. baseline)	0.55	0.32–0.96	0.036

HAMD = Hamilton Depression Rating scale; BMI = body mass index.

Cigarette smoking, gender, PSQI score, and age at baseline were independent factors related to subjects with UI both at baseline and follow-up (Table 5).

**Table 5:** Factors independently predicting unintentional injuries both at baseline and follow-up.

	Odds ratio	95% Confidence interval	*p*-value
Cigarette smoking	11.89	2.77–51.16	0.001
Male gender	0.05	0.01–0.39	0.004
PSQI score	1.32	1.09–1.60	0.005
Age	1.12	1.03–1.22	0.01

PSQI = Pittsburgh Sleep Quality Index.

## Discussion

The GEE model demonstrated that the severity of depression was an independent factor related to UI at baseline and follow-up (Table 4). Compared with patients without UI, patients with UI had a greater severity of depression (Table 3). Among the different depressive states, patients with a current MDE had the highest percentage of UI, with more than half (56.1% and 52.4% at baseline and follow-up, respectively) of patients experiencing UI. At follow-up, the percentage of UI was significantly decreased. This may be mainly due to a decreased severity of depression, which might partially result from pharmacotherapy or self-remission of depression. The other three independent factors (age, BMI, and cigarette smoking) should not be causes of a decreased percentage of UI at follow-up for the following reason: Tables 3 and 4 showed that patients with a higher BMI, older age, and cigarette smoking were related to an increased percentage of UI. At follow-up, the mean age and BMI and the percentage of cigarette smoking of the full sample slightly increased. The changes in the three variables at follow-up had a trend toward an increased percentage of UI.

The result that depression might increase the risk of UI might partially result from the following reasons: 1) Impaired attention, concentration, and other cognitive functions due to depression might impair a patient’s ability to watch out and keep away from dangerous situations. Moreover, some depressive symptoms, such as psychomotor retardation, fatigue, and leaden paralysis, might cause slower responses to sudden dangerous events; 2) Sleep disturbance was found to be a factor related to UI in previous studies [19,20]. Table 3 shows that the subjects with UI had a significantly poorer sleep quality. Although PSQI score was not a significant factor related to UI in the GEE model, the impact of sleep disturbance on UI might be included in the severity of depression, because sleep disturbance is an important part of depression [14]. In fact, PSQI score was an independent factor predicting subjects with UI both at baseline and follow-up; 3) MDD patients with suicidal ideation might not be mindful of their safety. In fact, depression is related to risky behaviors, such as risky driving [21]. Our results demonstrated that subjects with UI at baseline had a higher probability of having a history of suicidal attempt as compared with subjects without UI.

Several factors were also related to UI: 1) Cigarette smoking was an independent risk factor related to UI. Smokers have significant dose-response excesses of injury death after controlling for other confounding factors [22]. The associations of cigarette smoking and UI might result from several reasons, including direct toxicity, distractibility, smoking-associated medical conditions, psychiatric comorbidities, and behavioral characteristics [23]. 2) A higher BMI was an independent risk factor related to UI. Previous studies have reported an association of obesity with intentional injuries [24,25]. 3) Old age is related to degeneration of cognitive and physical functions, which might increase the risk of UI. 4) Although medications did not appear in the GEE model, one study reported that several medications used to treat psychiatric disorders are related to an increased risk of falling among psychiatric inpatients [26]. Another study reported that depression remained a risk factor related to UI after controlling for current antidepressant use and other factors [7].

There are two points worthy of note: 1) At baseline and follow-up, 40.4% and 27.7% of patients had at least one UI in the past one year, respectively. Approximately half of these patients needed treatment for UI (56.1% and 54.5%, respectively) and had functional impairment due to UI (49.1% and 45.5%, respectively). Therefore, UI were common and resulted in a greater burden in terms of medical care and society in general among outpatients with MDD; 2) Falling down was the most common injury in this study. In fact, depression is associated with an increased risk of falling in older adults because of gait unsteadiness [10,27].

Several limitations should be addressed. 1) In this neutral clinical study, subjects were treated with different medications and dosages. The total impacts of these medications on cognitive and motor functions were unable to be calculated. Although medications did not appear in the regression model, impacts of medications on UI were unable to be excluded. 2) This study was performed in a medical center. Expansion of these results to the general population should be performed cautiously, because some patients with MDD in the general population do not accept pharmacotherapy. 3) This study established some exclusion criteria. During the enrollment process, bias might have been introduced. 4) Subjects reported UI in the past one year. Memory bias due to depression might exist. 5) Owing to the limited budget for this study, systematic sampling was employed to select a small sample, which might limit the statistical power.

## Conclusions

UI were common among outpatients with MDD, especially in outpatients in a current MDE; therefore, UI should not be neglected. About half of the MDD patients with UI needed treatment and had functional impairment due to UI. A greater severity of depression, older age, cigarette smoking, and a higher BMI were identified as independent risk factors related to UI. The decreased percentage of UI at follow-up might result from improvement of depression. In clinical practice, physicians should highlight and educate patients about factors related to UI. Treatment of depression, control of body weight, and quitting cigarettes might be helpful for the prevention of UI.

## Supporting Information

S1 Dataset The dataset includes data related to unintentional injuries at baseline and one-year follow-up. (XLS)
